# Wear Resistant Coatings with a High Friction Coefficient Produced by Plasma Electrolytic Oxidation of Al Alloys in Electrolytes with Basalt Mineral Powder Additions

**DOI:** 10.3390/ma12172738

**Published:** 2019-08-27

**Authors:** Olga P. Terleeva, Aleksandra I. Slonova, Aleksey B. Rogov, Allan Matthews, Aleksey Yerokhin

**Affiliations:** 1 Nikolaev Institute of Inorganic Chemistry, Siberian Branch of the Russian Academy of Sciences, Novosibirsk 630090, Russia; 2 Department of Materials, The University of Manchester, Manchester M13 9PL, UK

**Keywords:** plasma electrolytic oxidation, friction coefficient, wear resistance, aluminum alloy, slurry electrolyte, basalt mineral

## Abstract

To achieve a better performance of engineering components, modern design approaches consider the replacement of steel with lightweight metals, such as aluminum alloys. However, bare aluminum cannot satisfy requirements for surface properties in situations where continuous friction is needed. Among the various surface modification techniques, plasma electrolytic oxidation (PEO) is considered as promising for structural applications, owing to its hard and well-adhered ceramic coatings. In this work, the surfaces of two Al alloys (2024 and 6061) have been modified by PEO coating (~180 µm) reinforced with basalt minerals, in order to increase the coefficient of friction and wear resistance. A slurry electrolyte, including a silicate-alkaline solution with addition of basalt mineral powder (<5 µm) has been used. The coating composition, surface morphology, and microstructure were studied using X-ray diffraction, scanning electron, and optical microscopy. Linear reciprocating wear tests were employed for the evaluation of the friction and wear behavior. It was found that the coatings reinforced with basalt mineral showed that the wear and friction coefficients reached values 10^−6^–10^−7^ (mm^3^ N^−1^ m^−1^) and 0.7–0.85, correspondingly (sliding distance of 100 m). In comparison with the characteristics of resin-based materials (10^−5^–10^−4^ (mm^3^ N^−1^ m^−1^) and 0.3–0.5, respectively), the employment of thin inorganic frictional composites may bring considerable improvement in the thermal stability, durability, and compactness, as well as a reduction in the weight of the final product. These coatings are considered an alternative to the reinforced resin composite materials on steel used in frictional components, for example, clutch disks and braking pads. It is expected that the smaller thickness of the active frictional material (180 μm) reduces the volume of the wear products, extending the service intervals associated with filter and lubricant maintenance.

## 1. Introduction

Using lightweight materials to reduce the weight of vehicles provides a potential solution to the topical problem of emission reduction in transport. Best industrial practices suggest using lighter aluminum instead of ferrous alloys. Aluminum alloys can have sufficient corrosion resistance and allow for up to 25–50% weight reduction in some parts [1]. However, because of their relatively low hardness and high friction coefficient, the parts made from Al alloys often have poor wear resistance, which limits their application range. While there are several coating techniques to harden the surface of aluminum parts, including hard anodizing, physical vapor deposition, and thermal spraying of hard coatings, the most promising technique appears to be plasma electrolytic oxidation (PEO) [2].

PEO is an electrochemical treatment, in which a high anodic potential leads to a dielectric breakdown of weak spots in the oxide film formed on the metal surface. The breakdown is accompanied by a localized energy injection, whilst leading to high temperature transformations of the coating materials and plasma-chemical reactions in the discharge channels. As a result, the obtained oxide layers are typically well crystallized and often possess high mechanical properties [2,3]. 

PEO coatings on aluminum are routinely used for sliding contacts in engineering applications (bearings and piston–cylinder pairs) [2,4,5,6], providing friction coefficients mainly between 0.02 and 0.4 (i.e., lower than or equal to that of an uncoated Al alloy) [7]. The lower end values of the friction coefficient correspond to the tests with liquid [8] or solid lubricants, such as graphite [9], tungstate [10], or polymers [11,12].

Although reports of high average friction coefficients exist, in many cases, the values measured for PEO coatings under dry sliding conditions range from 0.4 to 0.6 against various counterfaces [10,13,14,15], with the authors suggesting using these coatings on Al alloys in applications where weight saving is essential. Brake rotors with PEO coatings obtained from alkaline electrolytes provided satisfactory thermal stability, but a relatively low (0.25–0.3) coefficient of friction against organic-based friction materials (LowMet) [16,17]. Some reports quote friction coefficients as high as 0.8, but these values are usually observed in the beginning of the test, and subsequently drop to 0.5 [18], whereas in others, the sliding distances appear too short to accurately estimate whether the high values of the friction coefficient were able to remain in the long run [19,20]. More typically, the authors of reference [19] provided average friction coefficients for PEO coatings with thicknesses of 100 to 250 µm, as 0.64–0.68 and 0.68–0.86 versus bearing steel and tungsten carbide balls, respectively. In all of these studies, the coatings were obtained in silicate-alkaline electrolytes with no mineral additives; therefore, in addition to the aluminum oxides formed by substrate oxidation, they also contained silicate compounds formed by electrolyte components (e.g., silica or mullite). Furthermore, the authors of reference [19] provided the initial values of the friction coefficients for the uncoated alloy at 0.9 against the steel ball, and 0.88 against WC (tungsten carbide), while those at the steady state were 0.5 and 0.46, respectively. At the same time, the wear rate was as high as 4.5 × 10^−4^ mm^3^ N^−1^ m^−1^, signifying the fact that bare aluminum alloys are unsuitable for high friction applications. 

Usually, engineering components that require high friction (brakes and clutch elements) are made of polymer resin, with inorganic additions of asbestos, mica, talc, carbon fibers, metal wires, and powders [21,22,23]. The friction coefficient for such materials may vary between 0.3 and 0.5. Although cost effective and easy to manufacture, these materials feature some undesirable properties, including thermal instability, potential carcinogenic activity (resin and asbestos), and relatively high wear rates (10^−5^–10^−4^ mm^3^ N^−1^ m^−1^) [24]. The latter drawback is normally dealt with by employing friction pads with a high initial thickness (up to tens of millimetres), which constrains the miniaturization and simplification of the friction couple. Thus, it is of interest to combine the advantages of light aluminum alloys covered by hard coatings with stable values with a high friction coefficient and low wear rate in a one-step PEO process. 

A gradient structure of thick PEO coatings is known to cause considerable variation in friction coefficients, because the coating layers possess different phase compositions, microstructures, and properties [25,26]. In practice, the consistency (stability) of the mechanical characteristics during the component lifetime is quite important, especially in terms of the average values of the friction coefficient and wear rate. To control the mechanical characteristics of the PEO coatings throughout the operation, it was proposed to synthesize composite coatings with basalt mineral powder as a reinforcement additive. The reinforcement of the outer layer is important, as it is usually relatively soft and brittle. In addition, the outer layer must be thick enough to provide suitable durability. The inner layer provides load support and protection to the relatively soft aluminum substrate, whereas the interfacial barrier layer provides coating adhesion to the base metal. 

The mineral composition of basalt, which is a natural composite material, is defined by the microlites it consists of. These microlites are mostly composed of silicates, aluminosilicates, various silicon dioxides, and apatite. Its chemical composition may be represented as a set of metallic and non-metallic oxides with a variable content [27]. The water content in basalt can be as high as 10%, depending on its porosity and formation conditions [28]. It is known that solid materials may be incorporated into the PEO coating inertly (without considerable internal transformation), from slurry electrolytes in electrophoretic regime [29,30,31,32].

The goal of this study was to obtain ceramic coatings with high friction coefficients suitable for dry sliding, in order to avoid the disadvantages of conventional organic-based frictional materials. This paper discusses the original single-step PEO treatments of two examples among the most popular wrought series (20xx and 60xx) of aluminum alloys carried out in an alkaline-silicate electrolyte with additions of mineral powder, as well as general characteristics of the coatings with high and stable friction coefficients, which may offer new design solutions for clutch disk applications.

## 2. Materials and Methods 

Samples were made of two Al alloys, 2024 (AlCu4.5Mg) and 6061(AlMg), in the shape of a ring, with outer and inner diameters of 90 mm and 50 mm, respectively, and a thickness of 3 mm, providing a total surface area of 1 dm^2^. 

The electrolyte was prepared by adding basalt powder (130 g/L, particle size <5 µm) to an aqueous silicate-alkaline solution. The latter contained 10 g/L of technical liquid glass (SiO_2_/Na_2_O = 3.02; ρ = 1.41 g/cm^3^; dry residue 38 wt %) as a passivating agent, 7 g/L of sodium hexametaphosphate (Graham salt, CAS 10361-03-2) as a buffering agent, and 2 g/L of potassium hydroxide (CAS, 1310-58-3, >85%) to adjust the pH level to 11.5. The agarose gel (for electrophoresis, CAS 9012-36-6; 10 g/L) was used as a dispersant for the basalt powder.

PEO treatments were carried out in a 10-litre cylindrical stainless-steel tank equipped with a cooling jacket. The body of the tank served as the counter electrode. The process was carried out at constant temperature (T = 65–70 °C), with the electrolyte stirred constantly. A 50 Hz AC waveform with the initial average current densities of J_+_ = J_−_ = 10 A/dm^2^ was provided by a capacitor-type power supply. The duration of the PEO treatment was chosen at 90 min to provide the final coating thickness of 180 µm.

The coating thickness was measured by the “Quanix 1500” eddy-current thickness gauge (Quanix, Berlin, Germany) for the dielectric coatings. Vickers microhardness was measured using a PMT-3 tester (LOMO, St. Petersburg, Russia) on polished surfaces under a 1.96 N load. The microhardness values were averaged from ten indentations. The coating phase composition was studied using a DRON-3M X-ray diffractometer (XRD) (Burevestnik, Moscow, Russia) utilising Cu*_Kα_* radiation, with scans performed in the normal coupled mode in the 2θ range from 15° and 60°, at a rate of 0.02° per second. Phase identification was performed using a PDF-2 database. The surface microstructure and elemental composition were studied by Hitachi TM3000 Scanning Electron Microscope (Hitachi, Tokyo, Japan) equipped with an X-ray Energy dispersive spectroscopy (EDS) analyzer. Optical microscopy observations were carried out using a Neophote-2 (Carl Zeiss) metallographic microscope (Carl Zeiss, Jena, Germany) equipped with a 5 Mpx CMOS camera (Olympus, Tokyo, Japan). 

A linear reciprocating sliding ball-on-plate wear tester was used for the tribological tests, carried out under ambient atmospheric conditions against SAE 52100 bearing steel and WC-4%Co balls with a diameter of 6 mm at 5 N and 10 N normal loads, with a 10 mm stroke length (5 Hz), until the overall sliding distance of 100 m was reached. Other test conditions corresponded to ASTM G133-95. To evaluate the coating durability, the tests were performed on flat surfaces of the rings for four different residual thicknesses (180, 135, 90, and 45 μm). The three lower values were achieved by grinding the initial coating with abrasive paper up of to 4000 grit. After that, the samples were cleaned with acetone, washed with distilled water, and dried at 60 °C. The volume of the worn material was evaluated with a Dektak S3 profilometer (Bruker, Billerica, MA, USA) at a 7000 μm scan length with a ±1350 kÅ vertical limit. The mean average cross-sectional area of the wear scar was evaluated based on five measurements in random positions. 

## 3. Results

### 3.1. Basalt Characterisation

As the basalt composition depends on its origin, the samples of the powder material were analyzed by EDS and XRD before the PEO treatment. The results of the elemental analysis of the sample used in the experiments are presented in Table 1. The chemical composition of basalt can be expressed as a set of the following oxides: SiO_2_, Al_2_O_3_, MgO, Na_2_O, K_2_O, P_2_O_5_, CaO, TiO_2_, and FeO(Fe_2_O_3_). The elemental composition shows an excess of oxygen, caused most probably by hydration. 

**Figure 1:**
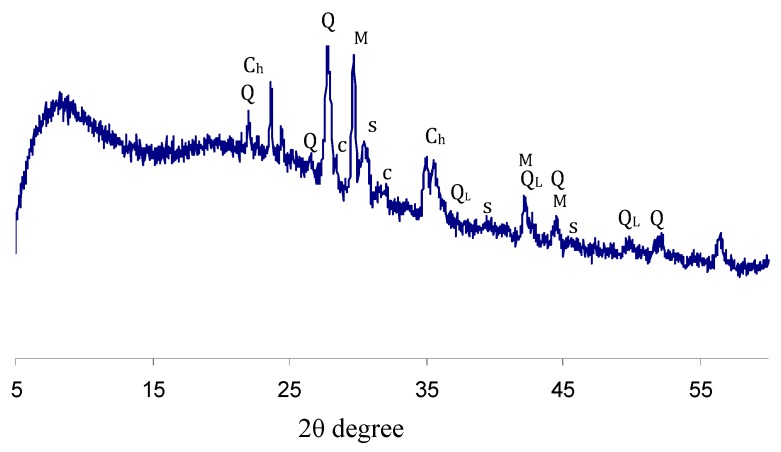
XRD pattern of basalt. Q—quartz; Q_L_—low temperature (SiO_2_); c—Cristobalite; C_h_—high temperature (SiO_2_); M—magnesium silicate MgSiO_3_; s—Stishovite (SiO_2_).

An XRD pattern of the basalt powder used is depicted in Figure 1. The XRD analysis shows that the sample contains high and low temperature phases of quartz. In addition, two phases of cristobalite (SiO_2_, low and high temperature), stishovite (SiO_2_), and magnesium silicate (MgSiO_3_) have been identified.

The expected oxides of aluminum, iron, calcium, titanium, or silicates have not been identified in the pattern that can be associated with (i) a substantial amorphous plateau between 5° and 45° 2θ, indicating that the major part of the basalt material is likely to be in an X-ray amorphous form, and in (ii) relatively small quantities. In addition, under Cu_Kα_ radiation, the mass absorption coefficient of iron is about 15 times greater than that of other elements, and, in this case, no traces of Fe could be found in the patterns. It is evident that the major elemental and phase compositions of the studied sample are typical of basalt mineral.

### 3.2. Coatings Structure and Composition

**Figure 2:**
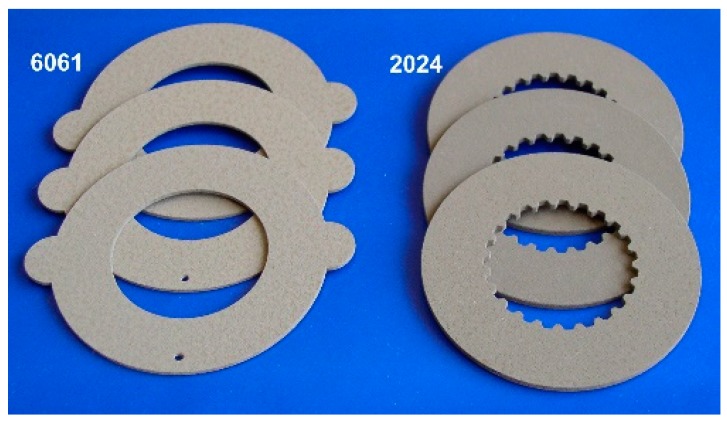
Appearance of the clutch disks with a plasma electrolytic oxidation (PEO) coating including basalt mineral powder.

**Figure 3:**
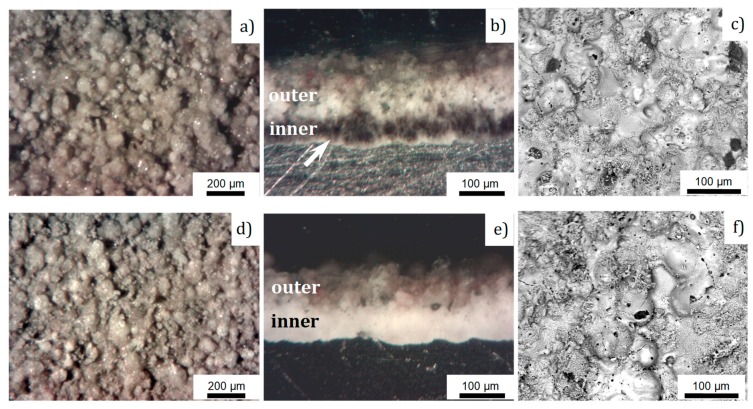
Optical micrographs of surfaces (**a**,**d**) and cross sections (**b**,**e**); SEM BSE images (**c**,**f**) of the PEO coatings obtained on the 2024 (**a**–**c**) and 6061 (**d**–**f**) aluminium alloys. The arrow indicates the interfacial layer.

The appearance of the aluminum parts with PEO coatings is shown on Figure 2. The total coating thickness of 180 μm was achieved for both of the substrate alloys. The surface plane optical micrographs of the obtained coatings and corresponding cross sections are presented in Figure 3a,d and Figure 3b,e, respectively. The surface morphology of the coatings on the A2024 and A6061 alloys is similar, featured by protuberances with a typical size of about 50–100 μm (Figure 3a,d). Moreover, the cross-sectional structures of the coatings on both alloys are similar, which is typical for PEO coatings on aluminum produced under an alternating current (AC; Figure 3b,e). In both cases, the outer and inner layers can be clearly distinguished. Moreover, on the samples of the 2024 alloy (Figure 3b), the inner layer appears to be divided into two sub-layers, as follows: a dark sub-layer in the middle part of the coating and a white sub-layer at the metal-oxide interface (marked with an arrow in Figure 3b), which is often referred to as an “interfacial barrier” layer. The inner layer always appears dark when copper is incorporated as one of the substrate alloying components. In the case of the 6061 alloy, the interfacial barrier layer has the same white color as the internal hard layer.

The backscattered electron images (BSE) of the coatings (Figure 3c,f) show pancake-like craters (30–50 μm) and a large number of small channels of breakdowns (<5 μm); detailed information about the typical morphology of the PEO coatings can be found elsewhere [33,34,35]. According to the data collected from the optical and SEM micrographs, there is no significant influence of the alloy composition on the morphology of the top coating. Therefore, we may assume that the proposed approach is suitable for different Al alloys, for which the composition is close to those of the 2XXX and 6XXX series. 

The results of the elemental analysis of the coatings are presented in Table 2. It can be seen that the surface chemical compositions of both of the coatings are similar. It is likely that Na and Si originate mainly from the electrolyte solution, whereas K, Ca, Fe, and P originate from basalt powder. According to the elemental analysis, the oxygen content exceeds the value calculated, based on the stoichiometry of the corresponding oxides (14–15%), which can be attributed to the presence in the coating of either hydroxides or adsorbed water.

**Figure 4:**
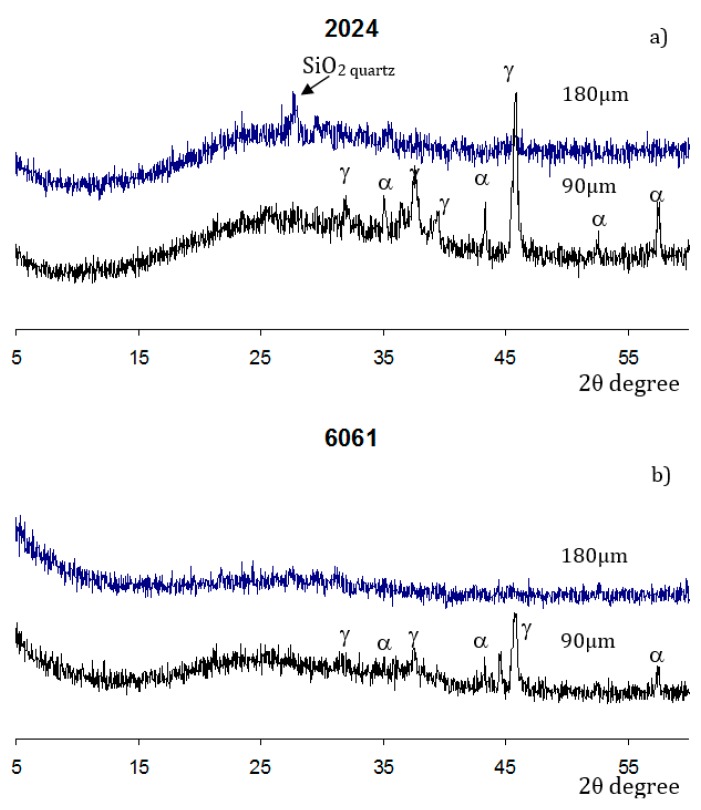
XRD patterns of PEO coatings on (**a**) the Al 2024 and (**b**) Al 6061 alloys, with two residual thicknesses (90 and 180 μm). α—α-Al_2_O_3_; γ—γ-Al_2_O_3_.

The phase composition of the studied coatings is presented in Figure 4. The XRD patterns taken at both the original coating, with a thickness of 180 μm, and the polished plane (90 μm), show a broad scattering between 15° and 35°, with a maximum at 25° 2θ, indicating that the major part of the coating material is X-ray amorphous.

The diffraction patterns of the outer coating layers on the studied alloys are slightly different. The magnitude of the amorphous halo is significantly higher for the coating on the Al 2024 alloy. Moreover, a noticeable peak of the high-temperature quartz phase can be seen in Figure 4a. Furthermore, a small peak corresponding to the γ-alumina phase can also be found. In contrast, the outer coating layer on the Al alloy 6061 is X-ray amorphous (Figure 4b). However, the inner regions of both coatings with residual thickness of 90 µm have a similar phase composition, comprising γ- and α-alumina. 

As the electrolyte solution includes noticeable concentrations of solid basalt particles and soluble silicate, the mechanism of the coating formation may comprise electrochemical substrate oxidation and electrophoretic precipitation from the electrolyte. The outer layer in the PEO coatings obtained from the alkaline-silicate solutions at a 50 Hz AC regime comprises typically ~1/3 of the total coating thickness [19,36]. In our case, the outer layer reached a half of the total coating thickness (see Figure 3b,e), confirming an electrophoretic mass transfer of basalt particles into the coating.

Two residual thickness values of 135 and 90 μm were chosen for the microhardness evaluation within the outer layer (incorporating basalt), and the third measurement was performed in the middle of the inner layer, containing α- and γ-alumina phases, at residual thickness of 45 μm (see Figure 3b). The microhardness values (as average of 10 indentations) at residual thicknesses 135, 90, and 45 μm were 1110 ± 320 HV, 1450 ± 210 HV, and 1850 ± 130 HV, respectively. It can be seen that the microhardness of the outer layer is significantly higher than that typically observed in the corresponding regions of the coatings obtained from alkaline-silicate solutions without particle additions (600–800 HV), whereas the microhardness of the inner layer is comparable to the typical values of 1900–2200 HV [8,36].

### 3.3. Tribological Properties

**Figure 5:**
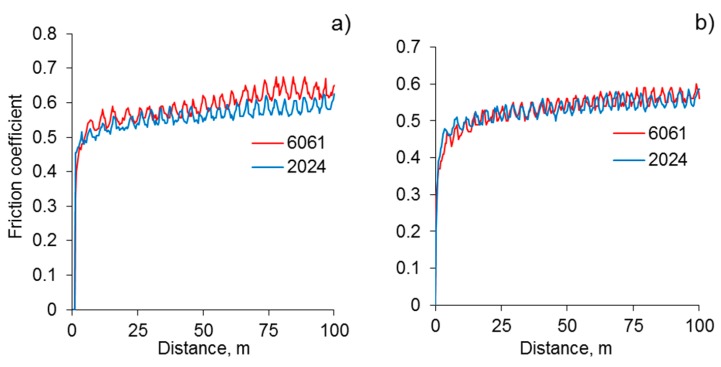
Evolution of the friction coefficient during the linear reciprocating wear test (LRWT) under a normal load of 5 N on the coatings with an initial thickness of 180 µm on the 2024 and 6061 alloys. The counterface materials were (**a**) bearing steel and (**b**) WC-4%Co.

The evolutions of the friction coefficient under linear reciprocating wear test (LRWT) conditions for the original coatings on the Al 2024 and 6061 alloys during the 100 m unlubricated sliding against both SAE 52100 bearing steel and WC-4%Co balls are depicted in Figure 5. The friction coefficients measured on the coated samples obtained under identical conditions demonstrate similar behavior, in a way, in that their values are rather high at 0.55–0.60 and remain constant or increase slightly throughout the test. This may be accounted for by an increase in the contact area between the coated surface and ball counterface as a result of progressive wear during reciprocal sliding. The observed similarity in the frictional behaviors could be attributed to the interaction of the counterfaces with the outer layer of the coating, and its composition and properties did not depend on the composition of the substrate (see Section 3.2).

**Figure 6:**
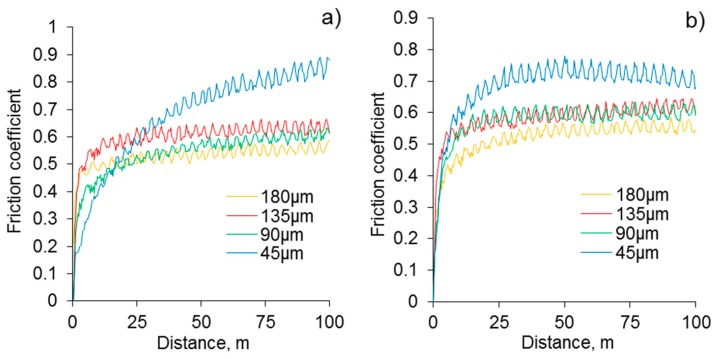
Evolution of friction coefficients during LRWT of PEO coatings with different residual thickness (180, 135, 90, and 45 μm). Normal loads are (**a**) 5 N and (**b**) 10 N. The counterface material is WC-4%Co.

As the coating morphology, elemental, and phase compositions are identical for both Al alloys, only the Al 2024 alloy underwent the tribological tests in further detail. The tests of the coated 2024 alloy with different residual thicknesses, against the WC-4%Co counter face under 5 N and 10 N loads (see Figure 6), have shown that the friction coefficients of the original coating (180 µm) are minimal (0.45–0.55); whereas at a thickness of 45 µm, the coefficient reaches a maximum in the range of 0.75–0.85. The values of the friction coefficient at the residual thicknesses of 90 and 135 µm are similar, and vary from 0.55 to 0.65 (see Figure 6a,b). As the surfaces of the coatings with a residual thickness of 180 and 135 µm are situated within the outer layer, and the residual thickness of 90 µm is situated within the intermediate region between the outer and inner layers, similar results for the friction coefficient values are not surprising.

**Figure 7:**
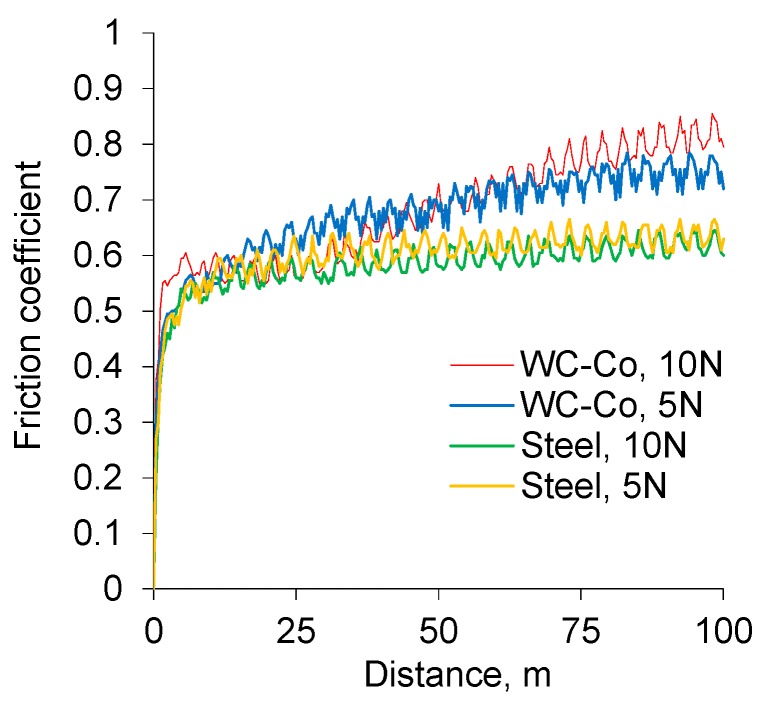
Evolution of the friction coefficient for PEO coatings with a residual thickness of 135 μm during LRWT at various normal loads (5 and 10 N) and against different counterface materials (WC-4%Co and bearing steel).

Figure 7 illustrates the variation of the friction coefficient depending on the employed counterface material. The increase in loading from 5 to 10 N did not lead to noticeable changes in the friction coefficient, however different counterface materials showed a variation in final values from 0.60 to 0.85 (for 135 µm residual thickness). 

**Figure 8:**
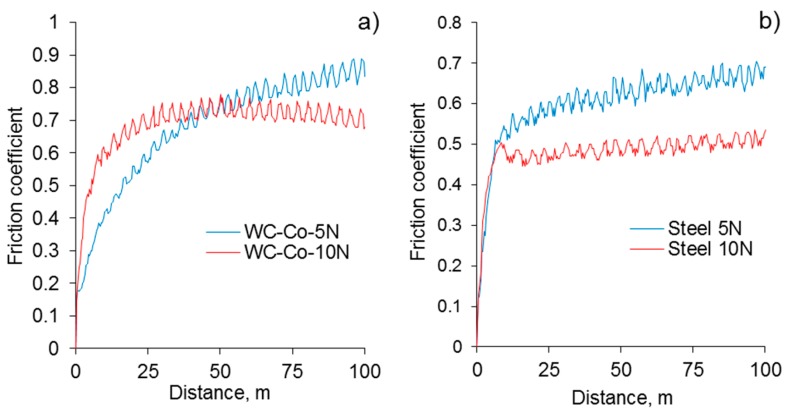
Evolution of the friction coefficient of the PEO coating with a residual thickness of 45 μm at normal loads of 5 and 10 N. Counterfaces are (**a**) WC-4%Co and (**b**) bearing steel.

Moreover, for the WC-4%Co counterface, the final coefficient of the friction was noticeably greater at the end of the test. A similar behavior was observed for the coatings with a residual thickness of 90 µm. In contrast, once the residual thickness reaches 45 µm, sliding occurs on the surface of the inner layer enriched with α-Al_2_O_3_ (~2200 HV), demonstrating a different behavior of the friction coefficient (Figure 8a,b). With the load increasing to 10 N, a decrease of the friction coefficient in respect to that at the 5 N load was observed for both counterfaces, at a final distance of 100 m.

However, from Figure 8, it can be clearly seen that the counterface material does matter in this case. Sliding tests on the inner layer have shown the highest friction coefficient through all of the performed tests (up to 0.85 for WC-Co and 0.7 for bearing steel). As each residual thickness was achieved by grinding, the coating surface at 45 µm was relatively smooth, and was well adhered (200–300 MPa [8]) to the metal substrate with no additional particles in a sliding zone, which excludes abrasive wear mechanism in this case.

At the normal load of 5 N, both of the counterfaces showed the same behavior as that for the higher residual thicknesses, although it should be noticed that the sliding against WC-Co corresponds to an extended initial breaking-in period (Figure 8a). Later, is likely due to the approximately equal microhardness of the inner coating layer and the WC-Co counterface. In contrast, the sliding of the relatively soft bearing steel against the inner coating layer could be accompanied by the fast plastic deformation of the steel ball surface, flattening of the contacting area, and the stabilization of the sliding conditions. 

Figure 8 illustrates that once the normal load increased to 10 N, the sliding conditions changed considerably. It should be noted here that in spite of the high hardness and wear resistance of the inner layer in the PEO coating, the interaction of the counterface and coating surface is influenced by the mechanical properties of substrate materials. It has been known [36] that the inner layer of PEO coatings includes hard grains of α-Al_2_O_3_ incorporated in a softer alumina-silicate matrix. Such structures possess a relatively low elastic module (10–40 MPa) compared with polycrystalline alumina (370 MPa) [37]. Thus, under the increased load (10 N), it is likely that the elastic deformation of the substrate beneath the coating takes place, considerably affecting the sliding conditions and values of the friction coefficient. These findings may be used as an estimation for the maximum working load of the designed component with a given residual coating thickness.

**Figure 9:**
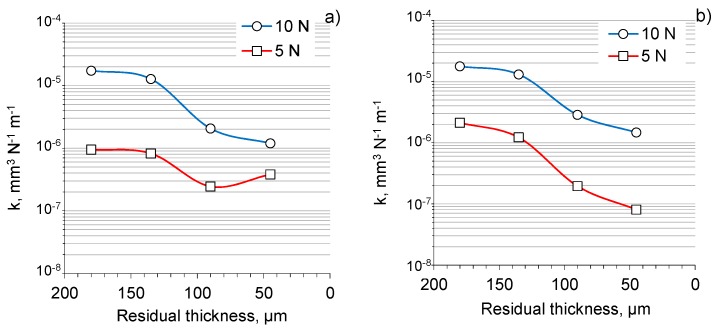
Wear coefficients (*k*) for coatings on THE Al 2024 alloy with different residual thicknesses derived from the results of the LRSW tests against (**a**) WC-4%Co and (**b**) bearing steel counterfaces at 5 and 10 N loads. The figure represents the averaged results, with the error bars being smaller than the size of data points.

The wear coefficients (*k*) derived from the results of the tribological tests for the coatings on the Al 2024 alloy with different residual thicknesses are depicted in Figure 9. The coatings showed wear coefficients of the order of 10^−5^ to 10^−7^ mm^3^ N^−1^ m^−1^, with a general trend to decrease across the coating thickness from the original surface (180 µm) to the inner layer (45 µm), which is in accordance with the pattern of the hardness evolution. The wear coefficients of the coatings tested against the bearing steel were slightly smaller than those obtained in the tests against tungsten carbide. In addition, the wear rates increased from the lower (5N) to the higher load (10N), probably because of the changes in the interaction mechanism.

Higher values of wear coefficients at the initial part of the tests (Figure 9) indicate that the coatings after PEO may require some post treatment in order to remove the roughest top part of the coating (Figure 3). However, this additional step is compensated by improved heat resistance, durability, and compactness, and a reduction in weight. Moreover, the smaller thickness of the active frictional material reduces the amount of wear products, thereby potentially increasing the service intervals accompanied by filter and lubricant maintenance.

### 3.4. Analysis of the Modified Layer Wear Mechanism

**Figure 10:**
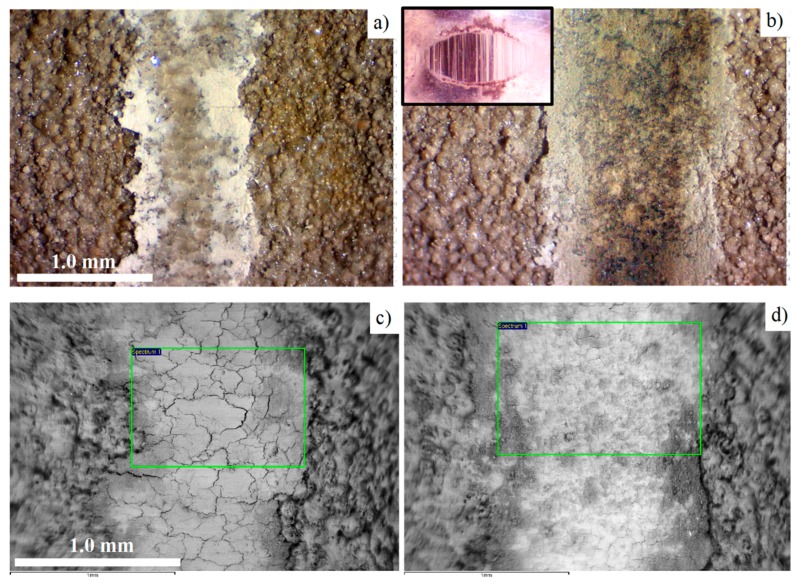
Wear scars on the PEO coating taken by optical microscopy (**a**,**b**) and by SEM BSE imaging (**c**,**d**) after wear tests against different counterfaces, as follows: (**a**,**c**) WC-4%Co and (**b**,**d**) bearing steel. Inset in (**b**) shows the bearing steel ball the surface after test. The rectangles on (**c**,**d**) designate the areas of the EDS analysis shown in Table 3.

As information about the friction and wear behavior of the inner dense layer of PEO coatings on Al alloys has already been widely reported (see references in Introduction), we will focus our attention to the wear mechanism of the upper-most coating part enriched by basalt powder. For these purposes, additional 18-meter wear tests against WC-4%Co and bearing steel counterfaces have been performed with a 10 N load, and with the same conditions as described in the experimental section. The optical and electron microscopy images of wear scars are depicted in Figure 10.

**Figure 11:**
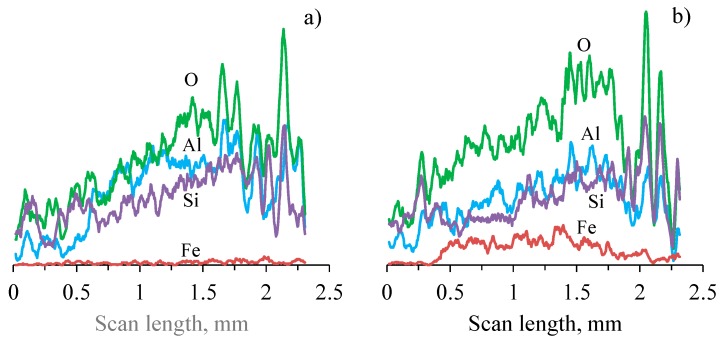
EDS analysis profiles (a.u.) for O, Al, Si, and Fe across wear scars on the PEO coating after different counterfaces for (**a**) WC-4%Co and (**b**) bearing steel.

The wear scar resulting from the interaction with WC-Co is noticeably narrower than for the bearing steel (Figure 10). The interaction of the basalt-enriched layer of the PEO coating and the bearing steel counterface is accompanied by the considerable transfer of ball material in the contact area. This is evident from the specific brown color of the wear scar (Figure 10b); the domination of the white areas in the SEM BSE image (Figure 10d), which is attributed to the presence of heavier elements; and from direct the EDS analysis of both rectangular areas (Figure 10d, Table 3) and line scans across the wear scars (Figure 11). In contrast, the wear scar developed in the test against WC-Co ball looks clean (Figure 10a,c), with negligible material transfer in the friction contact (Figure 10c, Table 3); neither tungsten nor cobalt contaminations have been detected by the EDS analysis.

**Figure 12:**
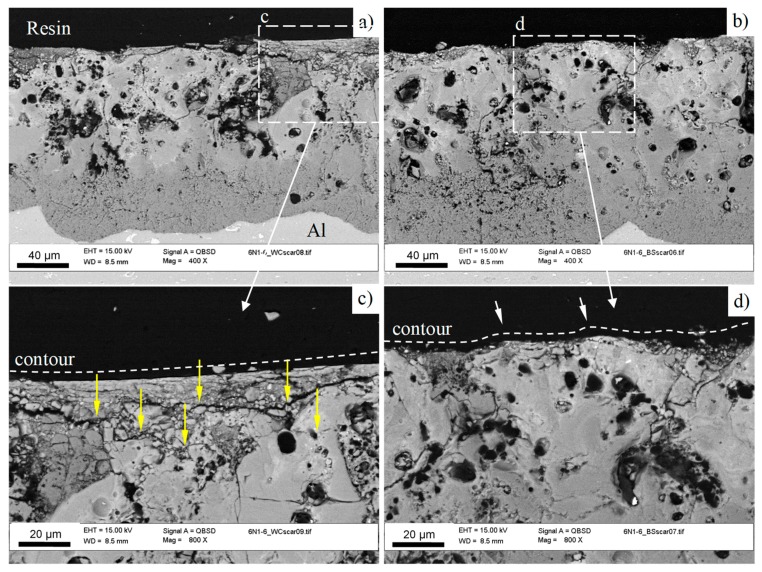
Cross sectional SEM BSE images of the bottom of the wear scars on the PEO coatings tested against different counterfaces of (**a**,**c**) WC-4%Co and (**b**,**d**) bearing steel. Yellow arrows denote delamination cracks.

Both wear scars demonstrate crack networks at the bottom of the sliding area; this is particularly distinguishable in the scar left by the WC-Co counterface (Figure 10c). To study the propagation of those cracks in depth, the coating cross sections have been investigated after wear tests (Figure 12a,b). It can be seen that the cracks are mainly concentrated in the 5−15 µm uppermost part of the coating, whereas the main coating thickness demonstrates only a few relatively deep cracks, typical for the PEO coatings tested against both counterfaces. At a higher magnification (Figure 12c), it is evident that the cracks tend to propagate in a horizontal direction (yellow arrows), causing further delamination of the coating fragments during the test against WC-Co. On the other hand, after the test against bearing steel (Figure 12d), the degree of defragmentation in the top layer is much smaller.

As shown in Section 3.2, the top layer of the PEO coating possesses an average microhardness of 780 to 1420 HV, attributed to the basalt and quartz particles incorporated into the relatively soft amorphous silicate matrix. Therefore, the wear mechanism for the studied counterfaces (tungsten carbide and bearing steel) is expected to be different, because of the noticeable difference of their microhardness (~2000 HV and ~850 HV, respectively) in respect to that of the coating.

Indeed, the cross sectional analysis of wear scars at a higher magnification revealed that the surface profile contour produced by the harder WC-Co ball is smooth, reproducing the shape of the ball curvature (Figure 12c), whereas after the softer bearing steel, the surface is wavy with noticeable asperities denoted by arrows in Figure 12d. As ceramic-like materials possess a very low capacity for plastic flow, the brittle fracture provides a significant contribution to wear, when the counter-face has a similar or higher hardness. This is exactly what was observed for the PEO coating interacted with the WC-Co ball, where fragmentation was present in both the surface plane and cross sectional directions.

In contrast, the interaction of the PEO coating with the softer bearing steel ball demonstrated an abrasive wear mechanism, including micro-cutting of the steel by the coating asperities, with intensive material transfer from the ball into the sliding area (Figure 11b and Table 3). This is evident from the micrograph of the ball surface after the test (Figure 10b inset), where scoring marks along the oscillations can be easily observed. However, the wear rate analysis (Figure 9) revealed that the top layer of the coating demonstrates similar wear rates against the WC-Co and bearings steel counterfaces (~1.8 × 10^−5^ mm^3^ N^−1^ m^−1^), despite the much lower microhardness of the latter. This could be due to the effect of the wear-induced debris acting as the third body in the tribological interaction with the bearing steel. The size of the debris is assumed to be in the submicron level, which is evident from the shining smooth surface of the scar on the bearing steel counterface, enveloping the scoring marks from the interaction with the coating asperities.

As the wear tests have been carried out at ambient conditions including water vapor and oxygen as the main corrosive agents, we can reasonably assume that the debris acting as the third body also included a noticeable amount of iron (hydr-)oxides, rather than metal particles, in addition to the removed coating material. Because of the large amounts of background oxygen in the PEO coating, we were unable to distinguish the iron (hydr-)oxides from the metal iron using EDS. However, from a comparison of the optical microscopy images (Figure 10a,b), the characteristic brown color of the wear scar on the coating tested against steel appears to support this assumption. 

Finally, we can provide a simplified estimation of the mean average contact pressure (P) developed at the end of the test, as follows:

(1) $$P\left[MPa\right]=\frac{F_{Load}\left[N\right]}{A_{contact}\left[mm^{2}\right]}$$

where *F*_Load_ = 10 N of the normal load, and *A*_contact_ is 0.418 or 0.698 mm^2^ for WC-Co and bearing steel, correspondingly. The average contact pressures were around 14 MPa for the bearing steel and 24 MPa for tungsten carbide. From Figure 6, it can be seen that after ~10 m of sliding distance, the tribological contact came to a steady state, so the obtained values of the contact pressures can be considered steady state too. Taking into account the typical loads in the clutch applications (0.05–5 MPa), the calculated values are three to four times greater, and the absolute values of wear coefficient are expected to be even lower than those demonstrated in our experiments. Therefore, the load-bearing capacity and wear resistance of the PEO coating reinforced with basalt powder can be considered sufficient to provide an alternative for frictional materials based on organic resins.

## 4. Conclusions

Thick (180 μm) PEO coatings with an outer layer reinforced by basalt mineral powder were fabricated on two commercial Al alloys, 2024 and 6061. For both of the alloys, the coatings showed a similar surface morphology featured by protuberances with a typical size of about 50–100 μm, and large numbers of small (<5 μm) breakdown channels. Although the coatings had a layered microstructure typical of PEO coatings produced in silicate-alkaline electrolytes under alternating polarization conditions, the incorporation of basalt particles into the outer porous layer resulted in a rather uniform distribution of the mechanical properties across the coating thickness. The Vickers microhardness values for the outer layer of the PEO coating were within 1110 ± 320 HV, which is noticeably higher in comparison to mulit layer without basalt reinforcement (700 ± 100 HV). With most of the coating material being X-ray amorphous, the presence of crystalline phases of α- and γ-alumina in the inner region located about 90 μm from the interface resulted in a hardness of 1850 ± 130 HV.

The PEO coatings on both of the Al alloys showed a similar tribological behavior, with friction coefficients recorded in the ranges of 0.60 to 0.85 and 0.50 to 0.70 against WC-Co and bearing steel counterfaces, respectively; these values are higher than those typical of reinforced resins (0.3–0.5). The coatings have shown a stable friction behavior, with wear coefficients being of the order of 10^−5^ to 10^−6^ and 10^−6^ to 10^−7^ mm^3^ N^−1^ m^−1^ for the outer and the inner regions of the coating, respectively, which is two to three orders of magnitude lower than for the resin-based materials. Moreover, the wear coefficient of the outer layer reinforced by basalt particles was almost independent on the counterface material, although different wear mechanisms were observed, including brittle fracture in sliding against WC-4%Co and abrasive three-body interaction against bearing steel.

Because if its inorganic nature, the coating material is expected to provide an improved heat resistance, durability, and compactness of friction components. The PEO coatings are thinner than conventional resin-based pads (180 μm versus 1–10 mm), which is expected to improve the thermal conditions of the sliding because of their lower thermal resistance and reduced weight, the and dimensions of the moving parts (e.g., because of their decrease in clutch/brake piston traveling distance). Therefore, the developed PEO coatings may be considered as an alternative to the frictional materials, based on the reinforced organic binding. 

## Figures and Tables

**Table 1:** EDS elemental analysis of the basalt mineral.

Element	O	Na	Mg	Al	Si	S	K	Ca	Ti	Fe
at. %	65.3	1.7	3.1	5.0	16.8	0.1	0.1	3.9	0.5	3.5

**Table 2:** Elemental composition of the surface layers on Al 2024 and 6061 alloys.

Element	O	Na	Mg	Al	Si	P	K	Ca	Fe
2024, at. %	65.9	6	1.1	7.6	8.8	5.2	1.7	1.6	2.1
6061, at. %	67.2	4.1	0.9	5.5	13.1	3.3	1.9	1.7	2.3

**Table 3:** Elemental composition of the wear scars for plasma electrolytic oxidation (PEO) coating for different counter bodies (BS—bearing steel 52100; WC—tungsten carbide 4%Co).

Element	O	Na	Mg	Al	Si	P	K	Ca	Ti	Cr	Fe	W
BS, at. %	69.0	4.2	0.7	8.2	6.7	4.3	1.3	1.0	0.1	0.1	4.3	0.0
WC, at. %	69.2	5.0	0.9	9.5	7.1	4.8	1.4	0.9	0.1	0.0	0.9	0.1
